# Food environments, food security and household food availability of circular migrant families: a mixed methods study among brick kiln laborers in Bihar, India

**DOI:** 10.1177/03795721231152057

**Published:** 2023-02-16

**Authors:** Reshma P. Roshania, Amy Webb-Girard, Aritra Das, Rakesh Giri, G. Sai Mala, Sridhar Srikantiah, Melissa F. Young, Tanmay Mahapatra, Usha Ramakrishnan

**Affiliations:** 1 Emory University, Atlanta, USA; 2 CARE India, Patna, India

## Abstract

**Background:**

Circular migration is the dominant pattern of movement in India, and is a livelihood strategy used by many food insecure rural households. Repeated shifts in food environments have important implications on household food security and dietary patterns, but have not been studied.

**Objective:**

To explore differences in the food environment, food security, and food availability between home and destination spaces.

**Methods:**

Mixed-methods research was conducted among circular migrant families working and residing on brick kilns in the state of Bihar. Utilizing stratified cluster sampling, two rounds of cross-sectional data were collected from 2564 families. Additionally, 25 in-depth interviews were conducted with circular migrant parents, kiln owners, and labor contractors. The Food Insecurity Experience Scale (FIES) was validated for use in our study population. Bivariate analyses were conducted to estimate the association of food insecurity with sociodemographic variables. Qualitative data were analyzed using descriptive thematic methods.

**Results:**

70 percent of respondents utilized at least one non-market source of food at the origin; at the destination, sources of food were limited to the private market. Despite higher food prices at the destination, perceived food affordability was higher during periods of migration, resulting in improved food security. Tubers, rice, and wheat were typically available in the household daily, whereas fruits, eggs, and dairy were typically unavailable during the week.

**Conclusions:**

Circular migration can enable short-term food security by improving food affordability. Policy frameworks must address the root causes of chronic food insecurity, especially among rural-to-rural circular migrant families.

## Introduction

The world’s highest burden of food insecurity is in South Asia, where over 740 million people are moderately or severely food insecure. ^1^ Within the region, India ranks lowest on the Global Hunger Index, ^2^ and is classified as facing serious hunger levels. 65 percent of India’s population resides in rural areas. ^3^ In the context of insufficient rural livelihood opportunities and rising agricultural uncertainties, migration of one or more household members is a means that many rural households utilize to cope with poverty and food insecurity. ^4^

The vast majority of migration within India is circular in nature, ^5^ cyclically undertaken for short periods of time with the intention of returning to the usual place of residence. ^6,7^ Circular migration is a strategy to diversify income-generating activities in more than one geographic space, often in addition to a small-scale agriculture; movement is, therefore, according to the agrarian calendar as well as seasonal labor requirements. ^8^ Official national statistics inadequately capture the magnitude of circular migration; informal estimates determine that 100 million people engage in circular migration within the country. ^6^ The poor, lesser educated, and those from socially marginalized groups such as Scheduled Castes and Scheduled Tribes are the most likely to engage in circular migration in India. ^8^

Research on the linkages between circular migration and food security in India has primarily focused on the food security impacts of remittances from male migrants on household members remaining in the place of origin. ^9^ In addition to unaccompanied male migration, an important stream of circular migration is the movement of family units. For example, the agriculture and construction sectors employ migrant women in high numbers, ^10^ and therefore represent destination sites for accompanying young children. The food security implications of circular movement for families who migrate together has not been studied in the Indian context.

Food security is determined by key aspects of the food environment – that is, the interface where the broader food system links with individual diets. ^11,12^ For circular migrant families, the food environment shifts episodically between home and destination spaces. The availability of foods, food prices, and time and distance to markets, can vary between home and destination as a result of differences in location and urbanicity; the ability to afford foods and time allocation for preparing foods can also alter depending on livelihood. Moreover, irrespective of geography, the very status of being a migrant prevents families’ access to state-led food security entitlements. The Public Distribution System (PDS) is a pillar of the country’s National Food Security Act, which recognizes the right to food by entitling below poverty line (BPL) households with subsidized staple grains. ^13^ Portability of PDS benefits was only recently launched in 2019, prior to which eligibility was based on domicile. However, rollout of the ‘One Nation, One Ration Card’ scheme has been slow and mired by administrative and technological challenges, as was evident during the first national Covid-19 lockdown when millions of migrants lost their livelihood and were facing difficulties in accessing rations. ^14^

The three ways of securing food with dignity – own production, purchase through livelihood income, and state provisions, ^15^ are thus all affected by migration, differentially at home and destination. This study aims to understand how circular migrant families experience these changes, and the multidirectional ways in which repeated shifts in their food environments influence household food security and food availability.

## Methods

### Conceptual Framework

The Agriculture Nutrition and Health Food Environment Conceptual Framework was used for the study design and analysis. ^11^ The framework distinguishes between the external and personal domains of the food environment. For example, in the external domain, availability is defined as the physical presence of foods within one’s food environment, whereas accessibility, a dimension of the personal domain, is determined by one’s ability to procure those foods based on distance and transport. Similarly, while food prices are established externally, affordability of food is a personal dimension based on purchasing power. We hypothesized that circular migrants experience shifts in both external and personal domains through changes in geography and livelihood as they move between home and destination.

### Study Setting and Design

The study was conducted among circular migrant families working and living on brick kilns throughout the state of Bihar. Among Indian states, Bihar has the lowest per capita income and the lowest worker population ratio, coupled with fragmented landholdings, inadequate agricultural inputs and climate volatility ^16^; it is no surprise, therefore, that Bihar experiences the highest circular migration in the country. ^8^ Brick kilns are major seasonal labor destination sites for circular migrant families. Labor contractors often recruit male-female pairs for many kiln occupations, thus resulting in the accompanying migration of young children. Brick kilns are mostly located in rural and peri-urban areas; migrant families reside on-site in rudimentary housing for the duration of the work season (during the dry months of October to June with some variation based on agro-climactic zone). This period is inclusive of the Rabi (winter) and Zaid (summer) agricultural seasons. Migrants who own land or work on land during the rest of the year return home for the start of the Kharif (monsoon) agricultural season.

Exploitation is frequent in India’s brick industry, due to the informal nature of work, as well as the methods of recruitment and payment ^17^. Lump sum advances are provided to workers by the labor contractor before the operating season, thus indebting families to the kiln owner. During the season, families are paid piece-rate, per 1000 bricks made as a family unit. This payment system invisibilizes women workers, and encourages long working hours; usually, the piece-rate wages earned amount to less than minimum wage when calculated per hour. Advances and other costs accrued such as emergency health care and weekly food allowances are deducted from a family’s earnings, which are withheld until the end of the season. On average, family units take home 46,000 rupees (USD 575). Often, the actual piece-rate wage is less than the agreed upon figure at the beginning of the season, and sometimes, if debts to the owner remain unsettled, families are bonded to return the following season. ^17^

The study was conducted using a mixed-methods approach. Quantitative survey data were used to examine sociodemographic and seasonal trends in food sources, food insecurity, and dietary patterns during migration among circular migrant families. Additionally, in the same study population, qualitative methods were used to explore how migrant families perceive and experience changes between home and destination food environments, and how these perceived changes in food environment potentially translate to changes in food security and dietary patterns.

The study was collaboratively implemented by CARE India and Emory University under the Bihar Technical Support Program partnership. Ethical approval was obtained from Emory University Institutional Review Board and the local review board, Ashirwad Ethics Committee. Verbal informed consent was obtained from each participant prior to the start of interviews.

### Data collection

#### Quantitative data

Two rounds of cross-sectional surveys were conducted in summer (June 2018) and winter (January 2019) to capture seasonal differences in food availability. June corresponds to the end of the migration season, reflecting an eight- to ten-month period since arrival to the kiln, whereas January is approximately two months into the migration season. Eligibility criteria for the study included 1.) self-identification as a circular migrant household, defined as living away from the home block (administrative sub-unit of the district) for employment purposes for a total of at least 60 days in the previous one year, with at least one return home in the previous one year; and 2.) presence of at least one child under three years of age.

Using a stratified cluster sampling design, eighteen brick kilns (cluster) were selected randomly from each district (stratum). Clusters were sampled from a district-wise list of operational brick kilns provided by the Bihar State Department of Mines and Geology. Based on *a priori* sample size calculations, per cluster, three households with children under three were randomly selected. The final sample size was 2564 households (1094 from the June 2018 round, and 1470 from the January 2019 round) from 1068 brick kilns. 37 of 38 districts had operational brick kilns and were included in the survey sample.

Enumerator trainings were held before each round of data collection. Surveys were administered in Hindi, and were pretested before the start of the study. After orienting the kiln owner or manager to the purposes of our study, a household survey was administered to the selected child’s mother. Household food security was measured using the Food Insecurity Experiences Scale (FIES) of the Food and Agriculture Organization (FAO), using the previous one year as the reference period. ^18^ The FIES is a psychometric, experience-based scale that measures the access dimension of food insecurity. The scale covers a range of food insecurity severity, from mild to severe experiences, and has been validated for use globally, enabling cross-country prevalence comparisons. Household food availability was quantitively assessed using a predefined list of 31 commonly cooked and packaged food items (listed in Appendix 1); the food list was developed by CARE India as a part of its monitoring and evaluation activities. Enumerators asked mothers to report the number of days (zero to seven) in the previous one week that each food was present and/or cooked in the household; this tool was administered only to households with children 0-23 months (n=1960). Enumerators collected all survey data digitally on tablets using the SurveyCTO software, a mobile data collection platform with in-built logic checks to minimize data entry errors. CARE supervisory staff also conducted back-checks with a random subsample of research participants to ensure data quality.

#### Qualitative data

For the qualitative component of the study, three districts – Patna, Rohtas and Gopalganj, were purposively selected based on agro-climatic zone and rural/urban status. Within each district, to avoid respondent fatigue, four kilns that were not included as a survey site for the quantitative study, were visited. Participants were recruited from each kiln using convenience sampling, while attempting heterogeneity with respect to community of origin, caste, family size and migration histories. Interviews were conducted in the late morning and early afternoon, which are rest periods of the working day in summer due to intense sun and heat.

An interview team of two female researchers and one male researcher conducted in-depth interviews using semi-structured interview guides with circular migrant mothers (n=11), circular migrant fathers (n=6), and key informants including labor contractors (n=2), brick kiln owners (n=2), and brick kiln managers (n=4). None of the selected migrant mothers and fathers were from the same household. We targeted to interview one migrant mother, and either one father or one key informant per kiln visited. Interviews with fathers were conducted by the male member of the team, interviews with mothers were conducted by the female members of the team; interviews with contractors, managers and owners were conducted by both male and female members of the team. Interviews were conducted in Hindi and were audio-recorded after obtaining informed oral consent from the participant. Recordings were transcribed verbatim in the Devanagari script. All interviews were conducted in June 2018, towards the end of the migration season.

### Analysis

#### Quantitative

Descriptive and bivariate analyses were conducted to explore sociodemographic characteristics, food sources, and household food availability. Wealth quintiles were created based on factor scores derived from principal components analysis of asset ownership. The list of assets is included in Appendix 2. For household food availability, food items were categorized into the following 12 food groups: rice, wheat, roots and tubers, pulses, green leafy vegetables, other vegetables, fruits, dairy, egg, meat/fish, snack foods, and sweets. Descriptive statistics were derived to assess seasonal differences in household food group availability between the summer and winter rounds.

We validated the FIES data for use in our study population by checking the Rasch model assumptions using the RM.weights R package. ^19^ The items in the FIES are as follows: During the last 12 months, was there a time when, because of lack of money or other resources: 1. You were worried you would not have enough food to eat? 2. You were unable to eat healthy and nutritious food? 3. You ate only a few kinds of foods? 4. You had to skip a meal? 5. You ate less than you thought you should? 6. Your household ran out of food? 7. You were hungry but did not eat? 8. You went without eating for a whole day? Although these items are in order of food insecurity severity according to the global reference scale, the FIES is grounded in item response theory, and thus the order of items may vary within different subpopulations based on differing coping mechanisms. We present relative item severity parameters, which are interval level measures, for this study population. The food insecurity raw score is calculated by adding the number of affirmative responses to each of the eight items in the scale. We defined food security as raw scores of 0 to 3 (inclusive of none and mild food insecurity), and food insecurity as raw scores of 4 to 8 (inclusive of moderate and severe food insecurity). We then examined bivariate associates between food insecurity and sociodemographic variables, stratified by season. Analyses were conducted in SAS 9.4; population characteristics and food security estimates considered the clustered sampling design, and therefore all bivariate analyses used the Rao-Scott chi square test. Alpha was set at 0.05.

#### Qualitative

The first author, who is fluent in Hindi, analyzed the written transcripts using *a priori* English codes based on the in-depth interview guides and the conceptual framework. Broadly, codes corresponded to food acquisition, food environment dimensions (availability, accessibility, prices, and affordability), food security experiences, and diet. Emergent codes were also identified through the process of transcript review and memo writing. Descriptive thematic analysis was conducted using MAXQDA 2018 software. Quotes presented in the manuscript were translated into English by the first author.

## Results

### Population Characteristics

#### Survey Sample Description

The majority of circular migrants in the survey sample were intrastate migrants (53%), followed by migrants from the neighboring state of Jharkhand (33%) (Table 1). Scheduled castes, almost half of the sample, were disproportionately less likely to own land and obtain PDS rations compared to other caste groups. The poorest circular migrants were least likely to own land for home production of food, and were also the least likely to obtain PDS rations – among those in the lower wealth quintile, 36% of respondents accessed PDS rations, compared to 63% of those in the higher quintile.

#### Qualitative Sample Description

All interviewed migrant mothers and fathers except one (intrastate migrant mother) were from Jharkhand, the majority of whom were from tribal communities. Migrant mothers and fathers had a range from one to five children. The youngest accompanying child was six weeks of age, and the oldest accompanying child was 13 years of age; the average age was 4.3 years. Most households on the kiln were nuclear family units; four migrant interviewees also migrated with parents, parents-in-law, or elder relatives to the kiln. Four out of eleven women interviewed were pregnant at the time of the interview. Both of the labor contractors interviewed were women from Jharkhand; one contractor also worked on kilns as a laborer. All of the owners and managers interviewed were men from Bihar.

### Food Environment

#### Food sources

**Figure 1:**
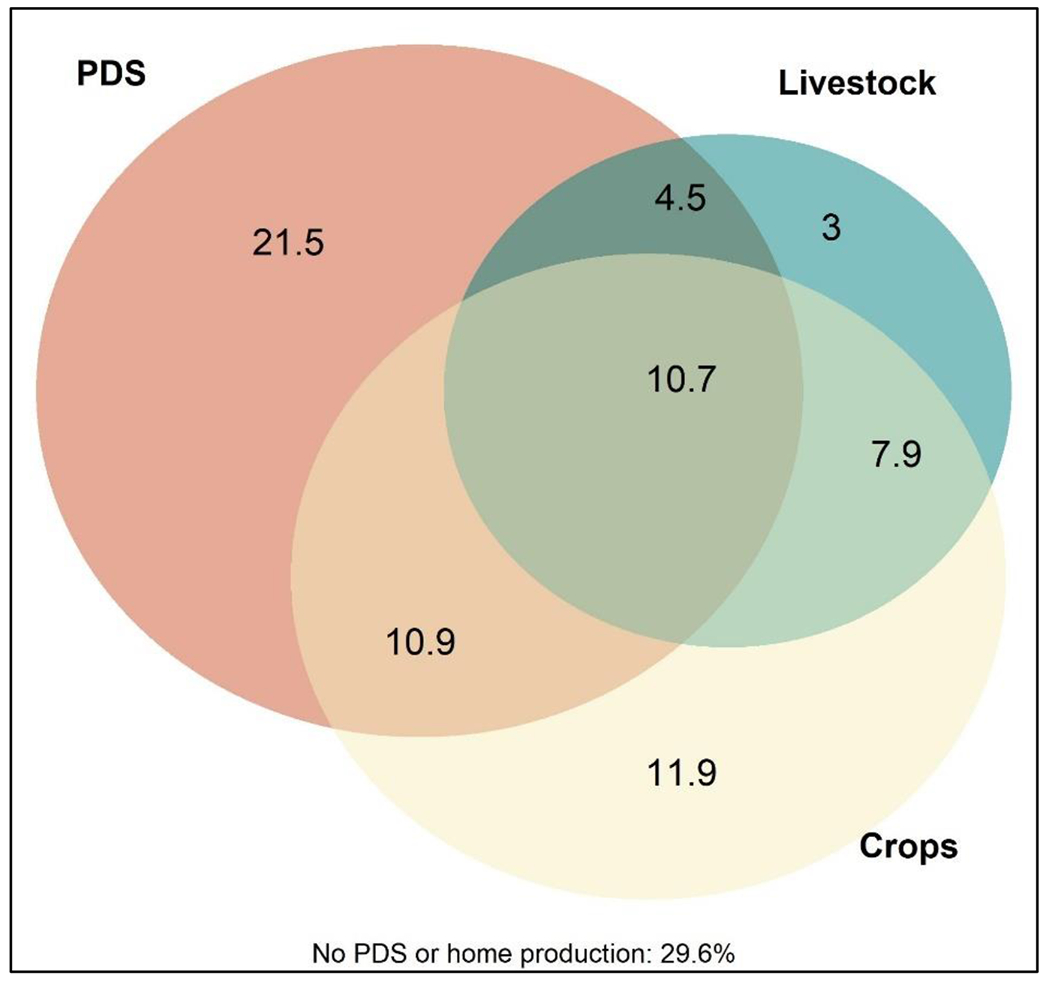
Non-market sources of food in place of origin (%), June 2018 and January 2019, n=2,564

Seventy percent of circular migrants from the survey sample utilized at least one non-market source of food while living in their place of origin (Figure 1). Around half of the respondents produced food for consumption, either by growing crops or raising livestock, or a combination of both. 26% of respondents reported both home production (crops and/or livestock) and PDS utilization as sources of food at home.

Qualitative findings demonstrate that sources of food differ considerably between home and destination. Most migrants (ten out of 17) included in the qualitative study owned some land that they used for farming and as a source of food while at home, corroborating survey findings described above. Lack of irrigation infrastructure was often discussed as a reason for engaging in circular migration during the dry months as reliance on rainfed irrigation means land can only be farmed during the Kharif agricultural season. One respondent, who owns eight acres of land said:

Where we live, all summer long, there is no irrigation. So, during the rains we farm, and during the summer we leave to earn. This is the only reason [we leave]. If there’s no irrigation what will we do all summer long? ‘Go somewhere and earn two paisa for savings’, we say and leave from home. (Father, Jharkhandi)

During the months when migrants are home, most households who farm rely on grains and vegetables that are produced for the majority of dietary needs.

When we stay in the village, we only have to buy salt. Everything else is there at home. Anything, green vegetables, garlic, onion. Even for oil we trade, we don’t buy. (Pregnant woman, Jharkhandi)

At the destination, however, sources of food are limited only to the private market for the several months families reside in the brick kilns.

Besides water, totally everything has to be bought. (Mother, Jharkhandi)

PDS rations are widely availed of at home as supplemental sources of food; though, four of eleven migrant mothers and fathers interviewed did not avail of PDS rations at home – among these non-users, one had been attempting unsuccessfully to apply for a ration card, and one participant had a BPL card, but rations in her village were only given to Antyodaya Anna Yojana card holders (issued to the poorest of the poor). Those who availed of PDS rations reported receiving mainly rice, wheat, kerosene and sugar.

Overwhelmingly, migrants noted they are ineligible for PDS while migrating, many referring to themselves as ‘pardesi’ (foreigners).

Here, nobody gets [government rations]. It would be nice if I received them, if everyone received them. We’ve come from a foreign land, what can we do? (Pregnant woman, Jharkhandi)

One kiln owner from Rohtas explained that laborers working on his kiln who are from the neighboring districts of Gaya and Aurangabad returned home once monthly to collect rations.

These people, one day of the month they go, the Biharis will definitely go home one day a month and release their rations from the quota shop. Rice, rations, whatever, they go and bring it back. It’s convenient because they go by train and the train fare is low. (Brick kiln owner, Bihari)

Wild foods, harvested from the jungle and common land, were another important source of food, especially indigenous leafy greens, mentioned by three migrants from Jharkhand. The sense of being an outsider at the destination, however, restricted this food source.

In our land, in our village, we can go to other fields and just take greens. Here where will we go? We’ve come to another land, what if someone says something? (Mother, Jharkhandi)

#### Food availability and accessibility

In qualitative findings on food availability, almost all migrant mothers and fathers expressed that the availability of foods in the market was largely similar between home and destination environments in terms of grains, vegetables, fruits and animal source foods. However, there were some exceptions, such as specific varieties of rice – while micronutrient rich parboiled rice was available and consumed at home, at the destination only raw rice was available.

While there is very little difference in the types of foods available between home and destination, some migrants expressed there is a greater *choice* to consume a diversity of foods in the destination. Households that produce food at home are largely restricted to consuming those foods which are grown. During migration however, all food must be purchased from the market. Some migrants discussed their ability to choose to purchase foods that are not grown at home, such as wheat, while they are migrating for work.

When we stay at home, we eat more rice and when we come here, we eat more roti. Here we buy and eat wheat. At home we don’t grow wheat nor do we buy it. If there isn’t money at home, how will we buy it? (Mother, Jharkhandi)

Food accessibility for circular migrants is shaped by the work schedule on the brick kilns; the weekly day off and disbursement of allowances were usually planned to coincide with the day of the haat (open air vegetable market). Distance and transport to market were also important aspects of food accessibility, and varied from site to site; sometimes transport to the market was provided by kiln managers on tractors, whereas sometimes migrants walked to the market. Accessibility to the market was a key factor in migrants’ experiences of working at a particular kiln, as demonstrated by the following quotes from two different women:

Laborers go wherever it is nice, where there is a bazaar, where there is a good market. Here the market is good. There are close to ten stores for vegetables, two to three stores for daal and rice. I don’t need to say anything. One laborer will go back home and tell others ‘that place was nice.’ (Mother and Labor Contractor, Jharkhandi)

This place is the worst. The bazaar, haat, everything is far. And the bricks are heavier too. (Mother, Jharkhandi)

Accessibility to markets is gendered; most migrant women reported their husbands go to the market on the weekly day off while they stay on the kiln, implying that gender norms around mobility and financial transactions persist across home and destination environments.

#### Food prices and affordability

While some migrants reported that food prices were the same at home and destination, others noted that prices of staples were considerably higher at the destination compared to prices at home.

There is a lot of difference. Here it is expensive. There it is cheap. Like potatoes. Here, potato is 18 rupees a kilo (USD 0.26). There, in our place, it’s ten rupees (USD 0.14). Ten rupees for two kilos. There is also a difference in rice. Here rice is 29 rupees, 30 rupees (USD 0.42). There it is 17, 18 rupees (USD 0.25). (Father, Jharkhandi)

However, many migrants indicated that despite higher prices in the destination, the affordability of foods is higher during migration. This is because of the regular weekly allowances that migrants received during the season for the purposes of buying provisions. Although these allowances were deducted from a family’s final earnings, which are withheld until the end of the season, the weekly sum of money enabled food affordability, especially for expensive desired items such as animal source foods.

Here, working, we can eat whatever we desire, meat, fish, eggs, kheer, roti, daal, puri, anyone can make and eat these things. If we want to eat chicken twice in a week, we can. Here, in a week, if we work or don’t work, even if we only work two days, we’ll still get 300 or 400 rupees (USD 4.28 – 5.71), so you can eat however much you desire. (Mother, Jharkhandi)

The ability to purchase desired foods during the migration season was also expressed by one of the labor contractors:

They eat less in the village. Here, they eat chicken, fish, eggs every day, these people. Here they earn, no? In the village they don’t earn, no? If someone wants to eat something, they’ll request 200 (USD 2.86) rupees from us. ‘Give us 200 rupees, we feel like eating chicken.’ They’re earning here so we give it. ‘Go and eat’. In the village they won’t get money immediately. In the village it’s tough. (Labor contractor, Jharkhandi)

While the perspective of most interviewees was that the weekly allowances provide a means to purchase sufficient and desirable foods during the migration season, two migrant mothers expressed that food is less affordable during migration, resulting in less dietary diversity. One mother made the distinction between receiving a weekly allowance versus the families’ full earnings:

We get more food at home. At home, we buy milk to feed the kids. Here, we can’t save enough, how will we buy? In vegetables, we only eat potato. We get eight days’ allowance – 800, 900 rupees (USD 11.43 – 12.86). Rations for five people, every eight days, don’t last the week. How will we get green vegetables? [At home] we earn and take the money. [Here] they give us allowances, we don’t get our earnings. Now when the kiln closes, then will we get earnings. (Mother, Bihari)

### Food Security

Findings from survey data demonstrate that overall, reported food insecurity in the previous one year was higher in the winter round compared to the summer round (Table 2). Food insecurity was negatively associated with education and wealth, and positively associated with household size at the kiln, across both seasons. Circular migrants who reported home production of crops were less likely to experience food insecurity in both summer and winter; those who reported raising livestock for animal source food consumption were also less likely to experience food insecurity, but this was only statistically significant in the summer. There was no significant association between PDS utilization and food insecurity in either season.

The validation exercise of the FIES data from our survey confirmed that the assumptions of the Rasch model were met. All item infit statistics were between 0.85 and 1.23, and outfit statistics were between 0.74 and 1.79. The Rasch reliability score was 0.72.

**Figure 2.**
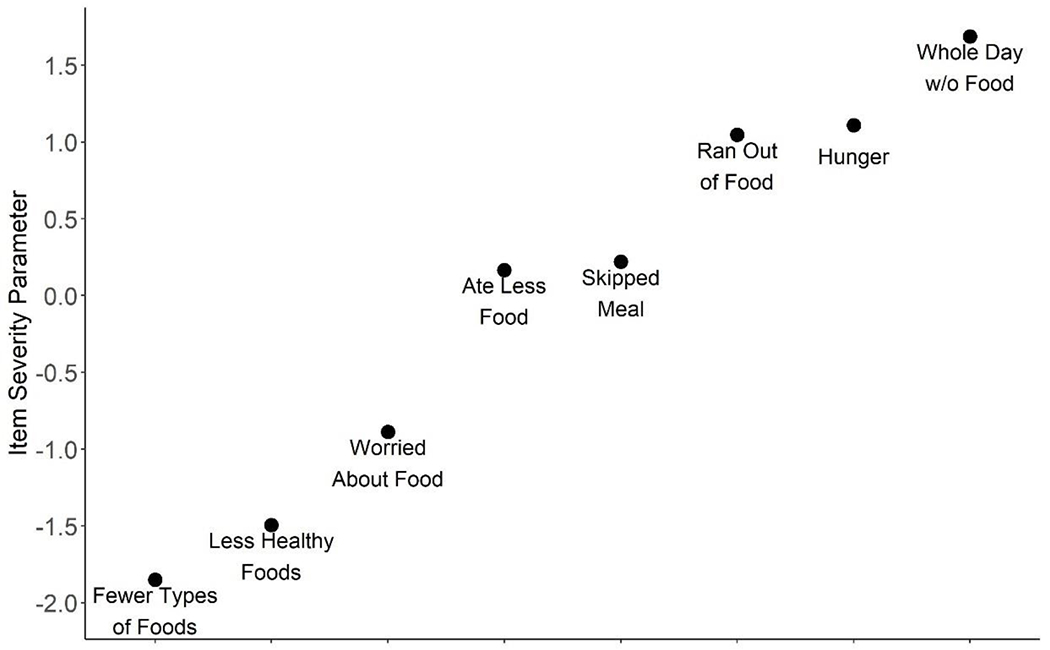
FIES item severity parameters, n= 2,564

Results from the relative item severity parameters are shown in Figure 2. On the continuum of food insecurity severity, the experience of consuming fewer types of food was the least severe among circular migrants, whereas anxiety or worry about running out of food occurred farther along the spectrum. The most severe experience of food insecurity was going a whole day without eating due to lack of money or resources.

Our qualitative findings indicated that livelihood for food security is an important driver of circular migration. Several migrant mothers and fathers expressed that they leave home every year to ‘fill their stomachs’. As described above, during the months spent away from home on the kiln, financial constraints to food are less compared to home because of regular weekly allowance disbursements.

Here, whatever we want to eat, it happens. At home, we have to think twice. (Mother, Jharkhandi)

The uncertainty of food acquisition at home also emerged as one reason for family members to migrate together as a unit.

[My wife and children] could have stayed at home, but then I would need to send money. The boss may or may not give money. What will they eat there? That’s why I brought them with me. (Father, Jharkhandi)

### Household Dietary Patterns

**Figure 3:**
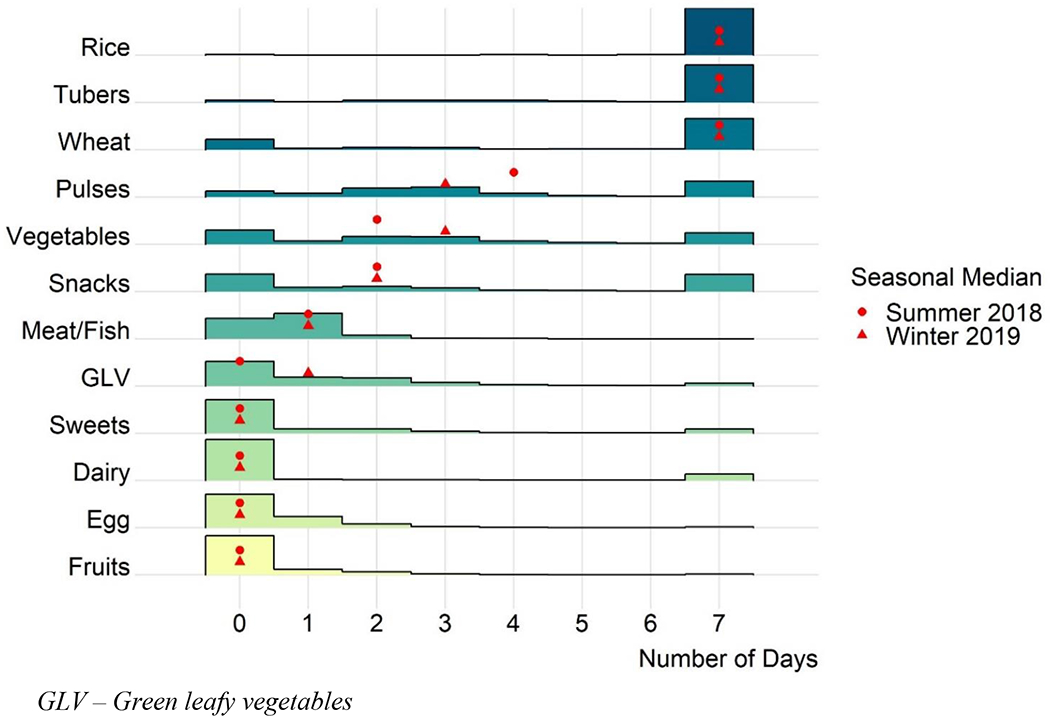
Histogram of number of days in the previous week food group was available in the home, June 2018 and January 2019, n=1,960

According to household food availability survey data, rice, tubers, and wheat were reported to be typically available in the household daily (Figure 3). Corresponding to seasonal availability, vegetables, including green leafy vegetables, were available more often in the winter compared to the summer (1.7 versus 0.8 days, respectively). Conversely, pulses were typically available 4.2 days per week in the summer on average, compared to 3.5 days in the winter. 82 percent of respondents reported dairy was not available during the previous week, and 79 percent reported fruit was not available any of the previous seven days.

Qualitative results support our survey data findings of daily rice and potato availability, and frequent availability of pulses. High consumption of rice was explained as a cultural preference by many migrant interviewees from Jharkhand.

We people eat rice, both times. We don’t feel full from eating roti. The people from here, they say ‘how do you people eat rice?’ We say to them ‘how do you people eat roti?’ They say ‘if we eat rice, we’ll feel hungry all day’. We say ‘if we eat roti, we’ll feel hungry all day’. We’ll never feel at peace. Everyone from Jharkhand, it’s our habit. (Mother, Jharkhandi)

Snack foods (mainly consisting of packaged biscuits and savory crisps) were typically available in the house two days of the week (Figure 3). Four mothers mentioned purchasing snack foods for their children in qualitative interviews. Some were aware of the unhealthy nature of certain processed foods. For example, one mother explained:

I get Horlicks (malt-based fortified sweetened drink powder) for [my son]. Before, those Kurkure (savory processed crispy snack) that are sold in all the shops, I used to feed him *a lot* of those. Then people told me, ‘Don’t feed him this’. So, I stopped, now I don’t feed those to him anymore. I mix Horlicks in hot water, add a little bit of sugar, and give it to him. (Mother, Jharkhandi)

The reasons for lower availability or consumption of green leafy vegetables during migration compared to while at home included the lack of affordability and the loss of access to wild greens as described above; in addition, one mother mentioned the lack of food storage in the summer heat coupled with once-a-week visits to the market as a barrier to purchasing green leafy vegetables.

If we get too many greens then they will dry out. Potatoes, daal, these things last. (Mother, Jharkhandi)

All interviewees except one reported purchasing and preparing chicken or fish once a week during migration, on their day off, as reflected in Figure 3. At home however, some migrants expressed animal source foods were eaten less often, only when there was money available to purchase them.

Meat, fish, if we get it [at home], we eat it. If we don’t get it, we don’t eat it. Only when there’s money can we eat, no? (Mother, Jharkhandi)

With respect to quantity of food, three migrant men shared they consume a greater amount of food and more often during migration because of the increased caloric demands from doing manual labor. Some women, however shared that they eat less often and less quantity while living on the kiln due to the workday schedule.

At home, we can sit and eat. Here, it’s as if we don’t have the right to eat. We eat, wash our hands, and get to work. Here, if we get to eat twice then we eat twice, otherwise, we don’t. At home, we eat a bit more. We sit around and can eat comfortably whenever we feel hungry. Here, after coming back from tiring work, what will we eat? (Mother, Jharkhandi)

## Discussion

This mixed-methods study explored how circular migrant families experience changes between home and destination food environments, and how these changes affect food security and dietary patterns during migration.

The majority of circular migrants utilize diverse non-market sources of food during the months residing in their home village. These sources include home production of crops and animal source foods, harvesting wild indigenous foods from common lands, trading, and government food entitlements. During the months residing at the destination, on the other hand, the only source of food is the private market. Findings demonstrate that a variety of food sources does not imply a greater diversity in food choice, purchase and consumption. For those who own land at home, the crops grown by the household largely define the food choices available. During migration for work in the brick industry, there is a reliable and consistent availability of cash, which allows families to purchase sufficient food and occasionally consume nutritious food groups that might not otherwise be affordable at home, such as fish and meat. While these cash advances are deducted from earnings at the end of the season, the regularity of income seems to enable food security or less severe experiences of food insecurity during the migration period for many families.

Results demonstrate that while the types of foods available and food prices were mostly similar between home and destination, circular migration, even within an industry known for exploitative practices, is an important strategy to improve food affordability. While for some, this may only be a temporary effect that allows for survival, for others it may be a pathway out of extreme poverty. This has important policy implications pertaining to the social protection of internal migrants. Despite the enormous contribution of internal migrants to the Indian economy, the government has historically adopted an unsupportive position towards migration. ^5^ Enactment of programs such as the National Rural Employment Guarantee Act (NREGA) intends to prevent distress migration from rural areas. Urban spaces remain hostile to migrants by failing to provide adequate and safe housing, water and sanitation facilities, education, and rations, further marginalizing groups that already face caste and class discrimination. The migration policy narrative centers around rural-to-urban migration. However, the concerns of rural-to-rural family migrants have been overlooked by policymakers. For those who choose to engage in circular migration for livelihood, migration must be made safer by enforcing existing labor legislation, such as the Building and Other Construction Workers Act, ^20^ that protects migrants from exploitation and outlines minimum wages, payment system requirements, safety standards, and provisions for employees, including on-site child care facilities and sanitation access, including in rural destination sites.

Similarly, in source villages, continued investments in agriculture, such as irrigation infrastructure, and strengthening implementation of rural employment and welfare schemes are important measures to ensure that migration remains a choice. Although PDS portability was officially introduced after our study period (thus migrants were not eligible for rations at the destination), due to targeting challenges, many eligible households were excluded from food security entitlements even in home villages; our results demonstrated those with relatively higher wealth were more likely to access PDS rations at home compared to those in the lowest wealth quintiles. This finding is consistent with prior analyses of BPL card distribution and utilization. Nationally, over 16 percent of the poor who are eligible for ration benefits are excluded from the program. ^21^ This figure is higher in many states of origin that were represented in our study, including Uttar Pradesh, Assam, and Jharkhand. In Uttar Pradesh, for example, 27 percent of the asset-poor do not possess a welfare card. ^21^ It is estimated that among those living in abject poverty, the poorest of the poor, exclusion is as high as 60 percent. ^22^ Circular migrants are more likely to be excluded from welfare schemes since BPL identification and targeting is based on population-based household surveys. Thus, the policy recommendation to employ multiple options to evaluate eligibility criteria for PDS rather than relying exclusively on BPL identification is especially relevant for circular migrant populations. ^22^

It is important to note that although circular migration may allow families to meet minimum energy requirements and diversify diets to an extent, it is far from likely that nutritional adequacy is being met. Findings from this study demonstrate that during migration, on average, cereals and tubers were available during most days of the week, whereas fruits and animal source foods were available less than one day of the week. The cost of the EAT Lancet recommended dietary intake for a healthy and sustainable diet for rural India is $3.33 per person per day for the least expensive foods. ^23,24^ This is over five times the actual amount spent on diets in rural India, which is $0.62 per person per day for the least expensive foods, and $1.00 per person per day for average priced foods. ^23^ Fruits and animal source foods, the most expensive food groups, represent the greatest share of the cost of the recommended diet, and the greatest deficits in actual diet spending. ^23^ Efforts to enable affordability of nutritious foods for all must be sensitive to the realities of migration. For example, interventions to diversify diets at the household level such as homestead gardening do not consider families who are away from their homes for several months of the year. Distribution of non-staple foods through PDS, coupled with effective implementation of portable entitlements is one potential migrant-inclusive way to improve access to affordable, nutritious foods.

Persistent low dietary diversity, both at home and during migration, may potentially explain FIES item severity findings. On the global scale based on FIES data from 121 countries including India, worry and anxiety about running out of food due to lack of money or resources ranks as the mildest experience of food insecurity. ^25^ In the context of this study however, worry about running out of food ranked more severe than eating fewer kinds of foods and inability to consume healthy foods. A possible explanation is that groups that typically consume a small number of food groups may not experience the lack of diverse or healthy foods as a coping response to food insecurity; rather, lack of dietary diversity and healthy foods may be experienced as the normal condition.

Due to the timing of the qualitative component, almost all of the interviewees recruited were from tribal communities in Jharkhand, where tribal land protection laws grant access to land for agriculture. Thus, a good representation of participants who do not have land for production of crops was not present in the qualitative sample, which is a major limitation to this study. Although overall, qualitative data were consistent with our survey findings, interviews with migrants from other communities would have enhanced our analyses.

From the survey data, the FIES reference period of one year is subject to recall bias, and additionally did not allow us to explore food insecurity specifically during the period of migration. We recommend further research to explore the validity of FIES measurement using relative time periods. Such research would be useful in other fields of research other as well, where experiences may mark changes in food security, for example, extreme weather events.

This study has several strengths. Rich insights were generated regarding the experiences of migrants with respect to changes in their food environments and food security through the utilization of mixed methodologies. Furthermore, while existing research has studied changes in diets and food security among rural-to-urban migrants, a substantial but understudied rural-to-rural stream was explored in this study. This allowed analysis of experiences of food environment shifts in the context of migration as a process, rather than changes due to urbanicity. Although the research was conducted in the brick kiln setting, findings may also be relevant in other industries, such as agriculture, where families temporarily migrate as a unit.

In conclusion, findings from the study suggest circular migration as an important strategy used by many poor households to improve food security and to some extent dietary diversity by enhancing the affordability of foods. It is important to recognize that these changes, while positive for some families, may be temporary, and not always lead to transformative upward mobility. While explicit inclusion of migrants in food security policy frameworks is necessary, for circular migrant families who migrate in the context of exploitative industries, upholding fundamental labor rights such as fair wages, safe housing and sanitation, and education of children is paramount in addressing some of the root vulnerabilities underlying chronic food insecurity.

## Figures and Tables

**Table 1.** Population characteristics of circular migrant households by land ownership and PDS utilization at home, June 2018 and January 2019, n = 2564

	All	Land ownership*	PDS utilization at home*
	n (%)	n (%)	n (%)

**Origin**			

Bihar	1370 (53.4)	259 (18.9)	590 (43.1)

Jharkhand	851 (33.2)	657 (77.2)	413 (48.5)

Assam	99 (3.9)	36 (36.4)	43 (43.4)

West Bengal	179 (7.0)	43 (24.0)	143 (79.9)

Other	65 (2.5)	22 (33.8)	31 (47.7)

**Caste**			

Scheduled caste	1159 (45.2)	269 (23.2)	532 (45.9)

Scheduled tribe	648 (25.3)	476 (73.5)	310 (47.8)

Other Backward Caste	600 (23.4)	225 (37.5)	282 (47.0)

General	157 (6.1)	47 (29.9)	96 (61.1)

**Religion**			

Hindu	2247 (87.6)	900 (40.1)	1039 (46.2)

Muslim	274 (10.7)	78 (28.5)	160 (58.4)

Other	43 (1.7)	39 (90.7)	21 (48.8)

**Respondent’s Education**			

No formal education	2177 (84.9)	780 (35.8)	1008 (46.3)

Up to 8th standard	273 (10.6)	156 (57.1)	149 (54.6)

Above 8th standard	114 (4.4)	81 (71.1)	63 (55.3)

**Wealth Index**			

Lower	509 (19.9)	49 (9.6)	185 (35.8)

Second	516 (20.1)	136 (26.4)	205 (40.4)

Middle	513 (20.0)	185 (36.1)	232 (45.2)

Fourth	514 (20.0)	270 (52.5)	274 (53.4)

Higher	512 (20.0)	377 (73.6)	324 (63.2)

**Total**	2564 (100.0)	1547 (60.3)	1220 (47.6)

* p < 0.05 for all variables

**Table 2.** Bivariate associations of food insecurity and sociodemographic variables by season, June 2018 and January 2019, n=2,560

	Food Insecurity Prevalence* (%, 95% Confidence Interval)			
	June 2018	*p-value*	January 2019	*p-value*
**Origin**				
Bihar	13.89 (10.73-17.05)		17.49 (14.79-20.19)	
Jharkhand	7.13 (4.54-9.72)		14.44 (10.18-18.71)	
Other	14.29 (4.70-23.88)	*0.005*	13.00 (8.53-17.47)	*0.211*
**Caste**				
Scheduled Caste	13.44 (10.13-16.76)		17.84 (14.76-20.93)	
Scheduled Tribe	6.46 (3.56-9.36)		13.36 (8.90-17.82)	
OBC	11.16 (6.39-15.94)		14.55 (10.94-18.15)	
General	18.75 (2.90-34.6)	*0.022*	15.57 (8.30-22.85)	*0.351*
**Respondent’s education**				
No formal education	11.87 (9.62-14.12)		16.55 (14.28-18.82)	
Up to 8th standard	6.60 (1.28-11.93)		13.25 (8.01-18.49)	
Above 8th standard	1.89 (0.00-5.56)	*0.036*	9.84 (2.25-17.42)	*0.234*
**Wealth index**				
Lower	18.60 (13.87-23.34)		26.64 (21.14-32.14)	
Second	12.21 (7.56-16.85)		17.63 (13.04-22.22)	
Middle	9.90 (5.60-14.19)		14.02 (10.23-17.81)	
Fourth	7.69 (4.16-11.23)		13.91 (10.03-17.78)	
Higher	4.48 (1.72-7.25)	*<0.001*	8.65 (5.31-12.00)	*<0.001*
**Household size at kiln**				
≤ 4	8.92 (6.38-11.46)		13.58 (10.95-16.21)	
> 5	13.11 (9.88-16.34)	*0.042*	17.84 (14.89-20.79)	*0.027*
**Crop production**				
No	13.64 (10.56-16.73)		17.57 (14.86-20.28)	
Yes	7.77 (5.32-10.22)	*0.003*	13.05 (10.14-15.96)	*0.024*
**Livestock for ASF consumption**				
No	12.79 (10.31-15.27)		16.94 (14.49-19.38)	
Yes	6.09 (3.29-8.89)	*0.001*	12.64 (9.11-16.17)	*0.057*
**PDS utilization**				
No	10.06 (7.66-12.47)		15.41 (12.73-18.09)	
Yes	11.92 (8.71-15.14)	*0.331*	16.37 (13.54-19.21)	*0.599*
**Total**	10.88 (8.84-12.92)		15.89 (13.81-17.98)	*0.001*

* Moderate and severe food insecurity

OBC – Other Backward Caste; ASF – Animal Source Food; PDS – Public Distribution System

## Appendix

**Appendix 1:** Table of food groups and corresponding survey items

Food Group	Item
1. Rice	1. Rice/poha
2. Wheat	2. Roti 3. Bread 4. Cerelac
3. Roots and tubers	5. Root vegetables (e.g. potato, taro)
4. Pulses	6. Lentils 7. Nuts (e.g. groundnut, almond, cashew) 8. Khichdi (rice and lentil mixture)
5. Green leafy vegetables	9. Green leafy vegetables (e.g. spinach, fenugreek)
6. Other vegetables	10. Other vegetables (e.g. brinjal, cauliflower)
7. Fruits	11. Vitamin A rich fruits (e.g. mango, papaya) 12. Other fruits (e.g. banana)
8. Dairy	13. Milk 14. Yogurt/buttermilk/paneer
9. Egg	15. Egg
10. Meat/Fish	16. Meat (e.g. mutton, chicken) 17. Fish
11. Snack foods	18. Maggi instant noodles 19. Kurkure 20. Biscuits 21. Cream biscuits 22. Rusk 23. Mixture 24. Bhuja 25. Nimki 26. Samosa 27. Daal/paneer pakoda 28. Vegetable pakoda
12. Sweets	29. Sweets (e.g. kheer, gulab jamun) 30. Chocolate/candy 31. Cake

**Appendix 2:** List of household assets to construct wealth index

1.	Electricity
2.	Mattress
3.	Pressure cooker
4.	Chair
5.	Cot/bed
6.	Table
7.	Electric fan
8.	Radio/transistor
9.	Black and white television
10.	Color television
11.	Sewing machine
12.	Mobile phone
13.	Other telephone
14.	Computer
15.	Refrigerator
16.	Watch/clock
17.	Bicycle
18.	Motorcycle/scooter
19.	Animal-drawn cart
20.	Car
21.	Handpump
22.	Water pump
23.	Thresher
24.	Tractor
25.	Gas stove
26.	Internet
27.	Air conditioner
28.	Cooler
29.	Type of toilet
30.	Type of water source
31.	Land
32.	Livestock
